# Response to comment on “Relation of fruit juice with adiposity and diabetes depends on how fruit juice is defined: a re-analysis of the EFSA draft scientific opinion on the tolerable upper intake level for dietary sugars” by Chen et al. 2023

**DOI:** 10.1038/s41430-023-01323-6

**Published:** 2023-10-18

**Authors:** Victoria Chen, Tauseef A. Khan, Laura Chiavaroli, Amna Ahmed, Danielle Lee, Cyril W. C. Kendall, John L. Sievenpiper

**Affiliations:** 1 Department of Nutritional Sciences, Temerty Faculty of Medicine, University of Toronto, Toronto, ON Canada; 2 Toronto 3D Knowledge Synthesis and Clinical Trials Unit, Clinical Nutrition and Risk Factor Modification Centre, St. Michael’s Hospital, Toronto, ON Canada; 3 Li Ka Shing Knowledge Institute, St. Michael’s Hospital, Toronto, ON Canada; 4 College of Pharmacy and Nutrition, University of Saskatchewan, Saskatoon, SK Canada; 5 Division of Endocrinology and Metabolism, Department of Medicine, St. Michael’s Hospital, Toronto, ON Canada; 6 Department of Medicine, Temerty Faculty of Medicine, University of Toronto, Toronto, ON Canada

**Keywords:** Metabolic disorders, Risk factors

## To the Editor:

We would like to thank Martínez et al. for their insightful comments [1] and for bringing to our attention the publication of the final version of the European Food Safety Authority (EFSA)’s Scientific Opinion on the Tolerable Upper Intake Level for dietary sugars [2]. We appreciate that the authors have included the EPIC-InterAct study [3] in the final version to reflect the totality of evidence and have concluded on fruit juice in general, rather than specifically on 100% fruit juice, to address the issue of misclassification. However, we note that an analysis separated by fruit juice type (100% fruit juice vs. non-specified fruit juice) is still missing. Although EFSA combined the analyses for 100% fruit juice and total fruit juice (including sugar-sweetened fruit juice) due to the similarity in their content of free sugars [2], this approach may not provide an accurate picture of the risk associated with each juice type. Large evidence syntheses of randomized controlled trials and prospective cohort studies have shown that the impact of fructose-containing sugars on cardiometabolic outcomes may depend on the food source [4, 5]. For example, harm is observed for sugar-sweetened beverages while benefit is observed for fruit. The beneficial nutrients and bioactive compounds found in natural fruit are often retained in 100% fruit juice but are either absent or present in only small amounts in fruit drinks. These nutrients and bioactive compounds may counteract any effect of free sugars in 100% fruit juice for cardiometabolic outcomes. For example, a recent systematic review and meta-analysis of controlled trials demonstrated that 100% fruit juice when providing less than 10% of calories decreased body weight and BMI, while fruit drinks increased body weight, BMI and body fat [6]. Similarly, systematic reviews and meta-analyses of prospective cohort studies have also demonstrated a benefit at low to moderate doses, showing a U-shaped association between 100% fruit juice intake and various cardiometabolic outcomes including hypertension [7], metabolic syndrome [4] and cardiovascular event risk [8]; however, this was not the case for non-specified fruit juice. Therefore, we emphasize the importance of conducting a stratified analysis by fruit juice type [9].

Martínez et al. also identified the low number and heterogeneity of the included studies as barriers to conducting a quantitative analysis. While we agree that more studies are needed to improve the certainty of the evidence, two studies are considered sufficient to perform a quantitative meta-analysis [10]. We addressed some of the heterogeneity by conducting separate analyses for children and adults, pooling only data that assessed the same outcomes (e.g., incident abdominal obesity was reported separately from change in body weight) and adjusting for the study period (e.g., studies including data on change in BMI over a study period different than 1-year were adjusted to per 1-year). We also provided separate conclusions based upon these populations and endpoints. Although EFSA’s final version of the scientific opinion on fruit juice has greatly improved from the draft version, our comprehensive and granular analysis based on fruit juice type provides additional information that is not present in EFSA’s final version. Therefore, our perspective piece should be seen as complementing EFSA’s scientific opinion and not detracting from it. Our study stands as a more comprehensive analysis of the work done by EFSA when relating to fruit juice type and adiposity and diabetes outcomes.

## Data Availability

All data generated or analyzed during this study are included in this published article.
